# A Smartphone App–Based Mindfulness Intervention for Cancer Survivors: Protocol for a Randomized Controlled Trial

**DOI:** 10.2196/15178

**Published:** 2020-05-11

**Authors:** Utkarsh B Subnis, Norman AS Farb, Katherine-Ann Laura Piedalue, Michael Speca, Sasha Lupichuk, Patricia A Tang, Peter Faris, Mark Thoburn, Bechara J Saab, Linda E Carlson

**Affiliations:** 1 Department of Oncology University of Calgary Calgary, AB Canada; 2 Department of Psychology University of Toronto Mississauga Mississauga, ON Canada; 3 Department of Community Health Sciences University of Calgary Calgary, AB Canada; 4 Mobio Interactive Toronto, ON Canada

**Keywords:** mobile health, psycho-oncology, mindfulness, mind-body therapies

## Abstract

**Background:**

Cancer patients transitioning to survivorship after completing cancer treatments need psychosocial interventions to manage stressors such as anxiety, depression, and fear of cancer recurrence. Mindfulness-based interventions (MBIs) are effective for treating these symptoms; however, cancer survivors are often unable to participate in face-to-face interventions because of difficulties such as work and family commitments, treatment-related side-effects, scheduling conflicts, and geography. Smartphone app–based MBIs are an innovative way to deliver psychosocial cancer care and can overcome several such difficulties, since patients can participate at their own convenience.

**Objective:**

The SEAMLESS (Smartphone App–Based Mindfulness Intervention for Cancer Survivors) study aims to evaluate the efficacy of a tailored app-based mindfulness intervention for cancer survivors (the *Am* Mindfulness-Based Cancer Survivorship—MBCS—Journey) for treating (1) symptoms of stress (primary outcome), as well as (2) fear of cancer recurrence, anxiety, depression, fatigue, and overall physical functioning (secondary outcomes). This is the first Canadian efficacy trial of a tailored mindfulness app intervention in cancer survivors.

**Methods:**

This is a randomized waitlist-controlled trial, which will evaluate the effectiveness of *Am* MBCS for impacting the primary and secondary outcomes in cancer survivors who have completed all their cancer treatments. Outcomes will be assessed using web-based surveys with validated psychometric instruments at (1) baseline, (2) mid-intervention (2 weeks later), (3) immediately postintervention (4 weeks), (4) 3 months postbaseline, (5) 6 months postbaseline, and (6) 12 months postbaseline. The waitlist group will complete all assessments and will cross over to the intervention condition after the 3-month assessment. In addition, data will be obtained by the smartphone app itself, which includes users’ engagement with the app-based intervention, their emotional state (eg, angry and elated) from a user-inputted digital emotion-mapping board, and psychobiometric data using photoplethysmography technology.

**Results:**

The study received ethics approval in September 2018 and recruitment commenced in January 2019. Participants are being recruited through a provincial cancer registry, and the majority of participants currently enrolled are breast (44/83, 53%) or colorectal (17/83, 20%) cancer survivors, although some survivors of other cancer are also present. Data collection for analysis of the primary outcome time-point will be complete by September 2019, and the follow-up data will be collected and analyzed by September 2020. Data will be analyzed to determine group differences using linear mixed modelling statistical techniques.

**Conclusions:**

Cancer care providers are uncertain about the efficacy of app-based mindfulness interventions for patients, which are available in great supply in today’s digital world. This study will provide rigorously evaluated efficacy data for an app-based mindfulness intervention for cancer survivors, which if helpful, could be made available for psychosocial care at cancer centers worldwide.

**Trial Registration:**

ClinicalTrials.gov NCT03484000; https://clinicaltrials.gov/ct2/show/NCT03484000

**International Registered Report Identifier (IRRID):**

DERR1-10.2196/15178

## Introduction

### Background

Previous research suggests that cancer survivors in Canada have several unmet psychosocial needs after completing treatments, which differ from patients newly diagnosed or undergoing treatment [1]. Almost half of all cancer survivors experience symptoms from late and long-term effects of treatments such as fatigue, pain, and distress [2]. Concurrently, they must deal with psychosocial stressors such as anxiety, depression, uncertainty about the future, and fear of cancer recurrence as they transition back to their previous roles and responsibilities at home and in the workplace; these factors can impair their quality of life, performance at work, and their ability to contribute to society [3,4]. Furthermore, the number of cancer survivors in Canada continues to rise due to rapid advances in early detection and treatments for cancer in an aging population. The most recent data suggest there are over 800,000 Canadians living with a history of cancer diagnosed in the previous 10 years [5]. Moreover, these numbers are expected to increase since survival data indicate 60% of Canadians with the top 4 most common cancer diagnoses are expected to survive at least 5 years postdiagnosis [6].

Similar trends of rising numbers of cancer survivors have been reported in the United States, and the world over [2]. Thus, cancer survivors globally can benefit from innovative interventions that address their unique psychosocial needs during survivorship. A growing body of evidence supports the efficacy of a range of mind-body therapies in alleviating these and other symptoms in cancer patients and survivors [7]. Among these therapies, mindfulness-based interventions (MBIs) have demonstrated significant efficacy in impacting psychosocial and physical health in the cancer population, such as the Mindfulness-Based Cancer Recovery (MBCR) program, a 9-week group behavioral treatment program that trains participants in mindfulness techniques through meditation and gentle movement practices [8].

The investigators on this research team LC and MS have studied MBCR for the past three decades and have tested its efficacy in a range of studies and groups of people with cancer, with success in impacting a range of biological and psychosocial outcomes including, but not limited to, symptoms of stress, quality of life, and mood disturbance [8-10]. This body of work on MBCR has spanned basic mechanistic research to clinical trials and implementation science. Most research studies of this nature have tested MBIs such as MBCR when delivered face to face in a group-based setting, although a variety of online-based and digitally adapted MBIs are now available to patients through smartphone apps [11]. Mobile app–based MBIs for cancer patients and survivors allow for considerable flexibility and appeal, especially since they eliminate the need for travel time and problems due to scheduling conflicts [11,12].

### Digital Health Interventions in Cancer Care

One of the most significant social and economic changes in the modern world has been the use of computer technology and the internet. Recent data indicate that 76% of Canadians now own a smartphone device with data connection across all demographic groups, and the numbers are projected to increase consistently [13,14]. The popularity of this medium is of great interest for health practitioners, researchers, and policy makers considering the wide-ranging capabilities of smartphone devices. In the field of cancer care, reviews of internet and smartphone app–based interventions for cancer patients and survivors have suggested that cancer patients find these interventions to be highly acceptable and feasible [15,16]. Furthermore, psycho-oncology researchers have advocated for more research with psychosocial interventions that can be delivered using the internet and smartphone apps in the cancer population [15-17].

### Benefits of App-Based and Online Mindfulness Interventions

App-based and online mindfulness-based interventions circumvent problems with traditional face-to-face delivery of MBCR such as work schedules, conflicts with other appointments, lack of childcare, and residing far from treatment centers in remote locations. Another potential benefit of app-based and online mindfulness interventions in cancer care is the considerable cost savings for the health care system without compromising on the quality of care, as online and artificial intelligence technologies can simulate the real-world experiences; in addition, studies have shown these interventions to be highly feasible and acceptable. For example, authors LC and MS conducted a feasibility trial of online MBCR, which found that more than 80% of participants completed the online MBCR program, and a 10% response rate to recruitment letters was achieved (the target was 5%) [18]. Also, recent systematic reviews and meta-analyses of app-based mindfulness interventions in the cancer population report high feasibility and acceptability; albeit engagement is an important variable to consider for intervention success [16,19,20].

However, while there are hundreds of commercially available mindfulness training apps, eg, *Headspace*, *Calm*, and *10% Happier* [21,22], only *Headspace* has been customized for cancer patients and been scientifically evaluated [21]. Data from a randomized waitlist-controlled trial and a prospective cohort study of *Headspace* have demonstrated good overall efficacy for improving outcomes such as quality of life and anxiety in women diagnosed with breast cancer [21,23]. However, the overall science of app-based MBIs is still in its early stages and far from achieving consensus about efficacy of intervention platforms, as well as understanding mechanisms of action [12,17]. For example, this is demonstrated by a randomized trial of *Headspace* with college students that reported no effects for the mindfulness component of *Headspace’s* app-based program [24].

Furthermore, *Headspace’s* audio content is exclusively voiced and delivered by the app’s founder in a monologue format [25]. This approach may not connect with all cancer patients and survivors, considering the importance of the patient-psychotherapist relationship in determining the success of any psychotherapeutic practice [26,27]. Also, cancer patients and survivors are more inclined to accept and utilize content created and delivered by clinical experts, for reasons such as source credibility and condition-specific content [28-30]. The research team for this study was interested in developing an app-based MBI for cancer survivors that simulated the interactive dialogic approach used in the MBCR program. The research team chose to evaluate the *Am Mindfulness* app as it could potentially address the gaps in the design and delivery of app-based MBIs for the cancer population.

*Am Mindfulness* is a readily editable digital platform with audio and visual capabilities and its content can be updated and modified with automatic app updates. *Am Mindfulness* was developed and is maintained by Mobio Interactive (MI) Inc. [31], a Canadian technology company based in Toronto, Canada, cofounded by MT and BS; user data are maintained on secure servers in Canada and meet security and Health Insurance Portability and Accountability Act privacy standards required for medical data. The name for *Am Mindfulness*, referred to simply as, *Am,* and pronounced “ahm” was chosen by MI for several reasons. First, “am” is the present tense of the verb “to be,” which is a linguistic embodiment of mindfulness given the high emphasis that mindfulness practices place on observing the present moment. Second, because of how “Am” is pronounced, it sounds similar to both the word for “soul” in French (“âme”) and the chant “ohm” used in ancient meditative practices from India. Finally, the name “Am” is relatively simple and less likely to bias individuals on the app’s purpose or offering in the way that names for other meditation-focused apps such as “Calm” or “Smiling Mind” may.

The design of *Am Mindfulness* allows for considerable flexibility with delivering content in real time through the “Journeys” app feature. Each app-based “Journey” involves a sequence of custom audio tracks, visuals with text and in-app exercises designed with a particular objective, eg, mindful survivorship. The content of a “Journey” can be seamlessly introduced and refined for a patient population in real time. MI describes their major motivation for creating the “Journeys” feature in *Am Mindfulness* as their commitment to create and deliver diverse content voiced over by clinical experts, in addition to mindfulness practitioners, to address the needs of specific patient populations [32]. Evidence from a recent randomized trial with a prerelease version of the *Am Mindfulness* app (called *Wildflowers*) has demonstrated efficacy evidence for reducing anxiety in college students before a stressful event of an examination [33].

**Figure 1:**
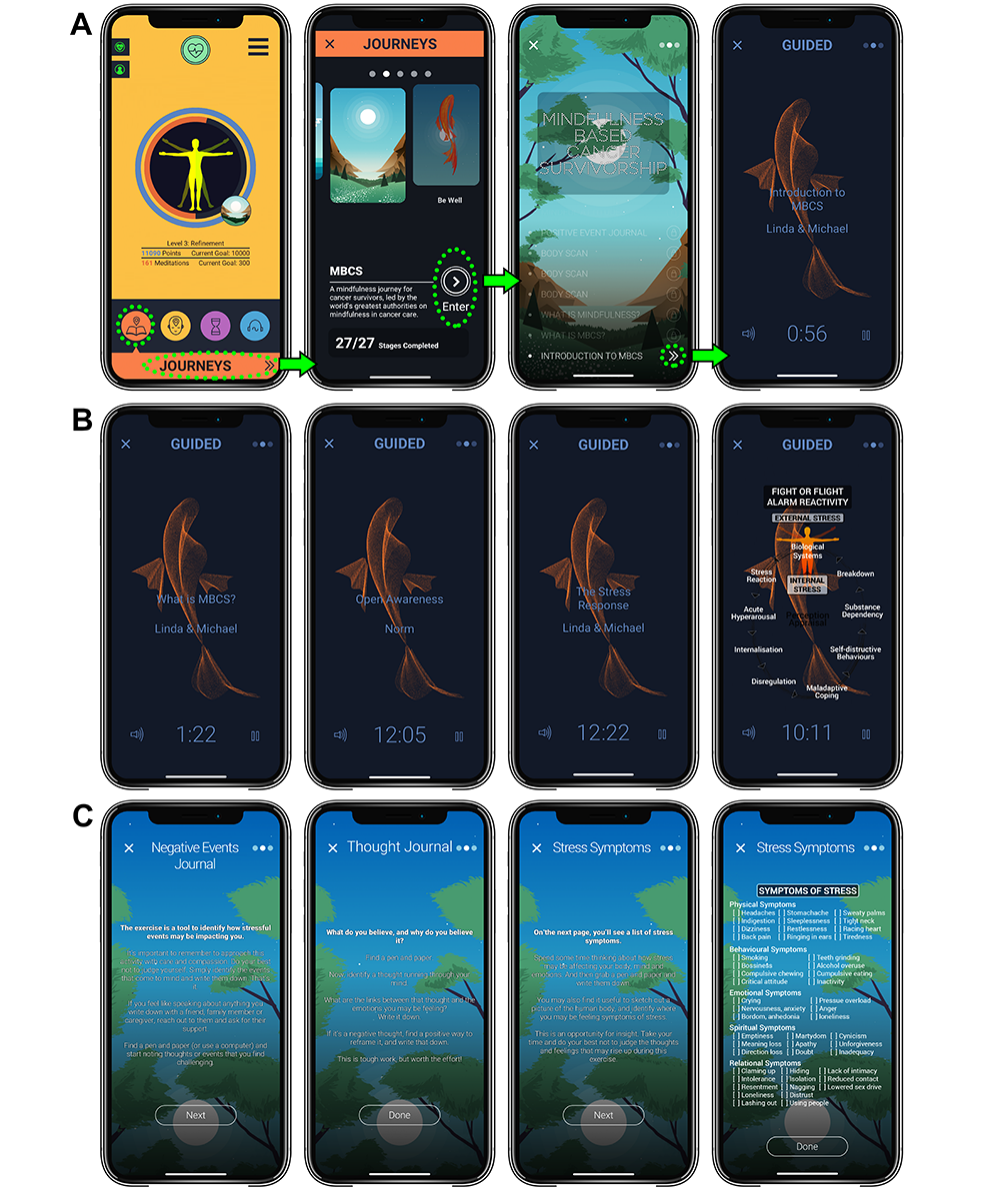
The Mindfulness Based Cancer Survivorship (MBCS) Journey Contained within *Am*. (A) Navigation menu. (B) Core audio content (C) In-app exercises.

In this project, the *Am* Mindfulness-Based Cancer Survivorship (MBCS) journey was developed and recorded in the voices of MBCR program facilitators LC and MS. Several audio tracks in the *Am* MBCS journey have a dialogue and interaction between LC and MS, who are clinical psychologists and leading experts on mindfulness-based cancer recovery, having developed and scientifically evaluated the MBCR program together for over 20 years. Details of the *Am* MBCS journey are described in the *Intervention* section and illustrated in Figure 1. In this study, we intend to test the efficacy of the *Am* MBCS Journey, referred to hereon as *Am* MBCS, to reduce stress in cancer survivors. As secondary objectives, we will investigate the feasibility of recruitment as well as the usability of the app and in-app data on specific usage patterns.

## Methods

### Study Design

**Figure 2:**
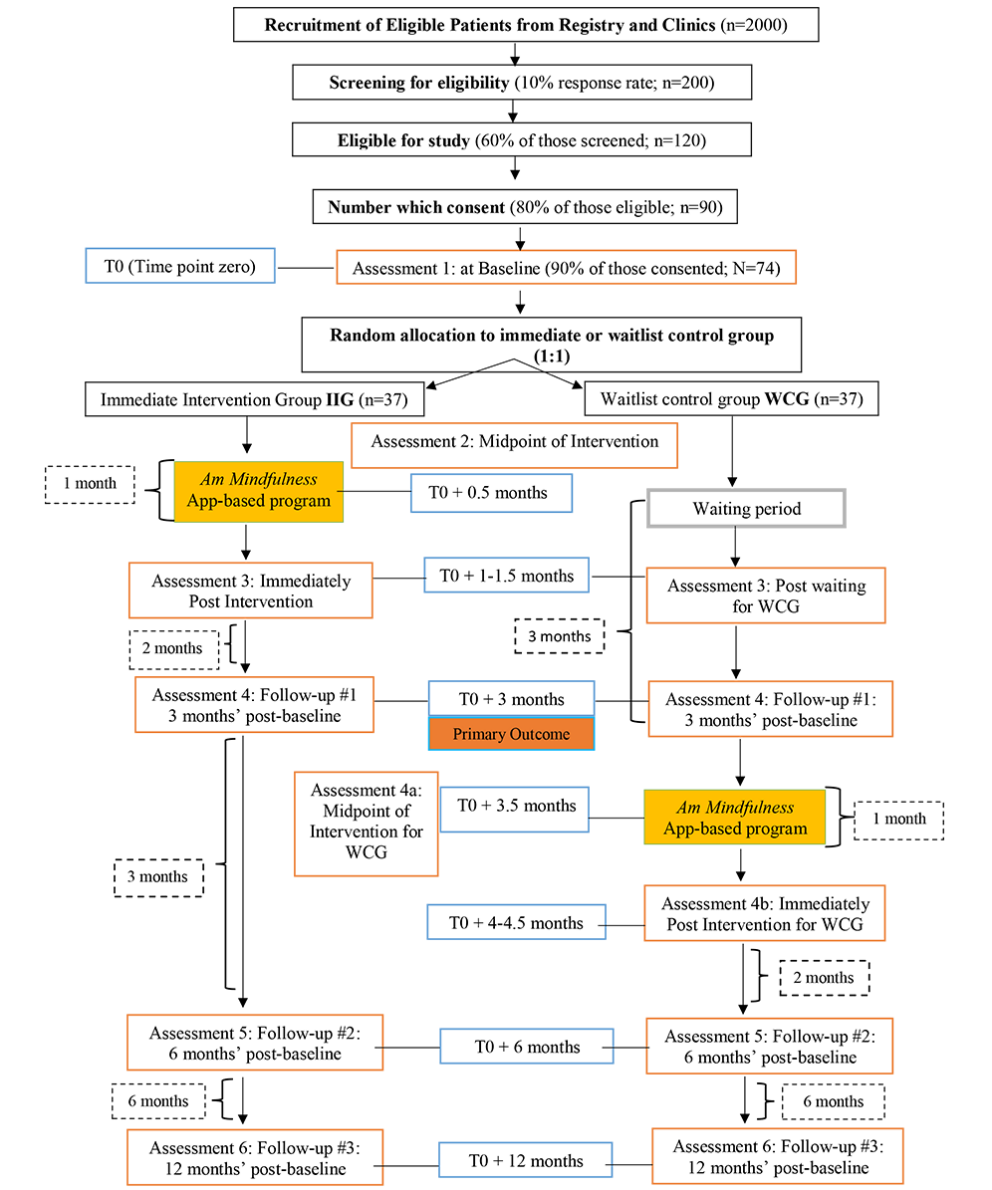
SEAMLESS Study Flow Chart. Includes study design, stage, and all time points of data collection for study assessments.

The study is a two-armed randomized waitlist-controlled design with 1:1 allocation to treatment (immediate *Am* MBCS app group) or control (waitlist usual care) arms, with assessments at six time points: (1) baseline, (2) mid-intervention (2 weeks later), (3) immediately postintervention (4 weeks), (4) 3 months postbaseline, (5) 6 months postbaseline, and (6) 12 months postbaseline. A detailed study flowchart is shown in Figure 2. This is an open-label trial, as blinding to interventions is most often not possible in psychosocial intervention research.

### Participants

Cancer survivors with any type of cancer who have completed their active treatments at least 2 weeks before recruitment in this study will be included. Inclusion criteria are intentionally broad to be pragmatic and improve generalizability to the real world. Refer to Table 1 for detailed rationale for each inclusion and exclusion criterion.

**Table 1:** Inclusion and exclusion criteria.

Criteria		Rationale and notes
**Inclusion**		
	(1) Men and women over the age of 18 years	Both men and women are included to broaden the generalizability of results and allow sex comparisons. All participants must be adults.
	(2) Completed all cancer treatments 2 weeks before enrollment	A brief period of time is required for patients to recuperate after their last treatment, before starting a new intervention.
	(3) Access to a smartphone with data connection	Patients will require access to a smartphone to participate. The study team will communicate primarily by phone, text message, and email. In case some patients’ do not have a data plan or an insufficient data plan with their smart phone, we will pay for their data connection (up to 0.5 GB/month).
	(4) Willing to give time for mindfulness practice	Patients need to have the motivation to devote approximately 20 to 30 min daily, which is equal to 5 to 7 sessions a week over the course of 1 month to do the mindfulness meditations and practices.
	(5) Sufficient ability to speak and read English	The audio lectures and meditations and assessments will be conducted in English, so participants must be able to understand the audio and fill out the questionnaires.
	(6) Willingness to be randomized into immediate or waitlist groups and complete all assessments	People must be comfortable with potentially having to wait to get access to the app-based program for another 3 months, as well as be motivated to give 30 to 40 min of their time to complete the online survey assessments.
**Exclusion**		
	(1) Suffering from current major depressive disorder, or other psychiatric disorder (self-reported) that would interfere with the ability to participate	Evidence indicates that participants with active psychological disorders should be first treated for these problems individually, before engaging in experimental mental health and meditation programs of this nature, which are not intended to treat these disorders.
	(2) Currently engaging in mindfulness meditation one or more times per week	To ensure sample homogeneity, the study will include participants who are NOT currently practicing mindfulness, using an app or otherwise. However, this would not exclude everyone who may have casually experimented with the aforementioned interventions in the past.
	(3) Cognitive impairment that would interfere with completing questionnaires or the intervention; <6 on the Brief Screen for Cognitive Impairment (BSCI) [3]	People require enough cognitive capacity to complete the questionnaires, navigate and listen to the app and complete homework independently. The BSCI only rules out those with significant cognitive impairment and will not exclude those with the milder cognitive impairment associated with cancer-related “brain fog.”

### Recruitment

Potential participants will be recruited at a comprehensive cancer center in Western Canada from a Provincial Cancer Registry.

#### Alberta Cancer Registry

The Alberta Cancer Registry (ACR) is a population-based registry, established in 1942, that records and maintains data on all new cancer cases and cancer-related deaths occurring in the province of Alberta. The registry records information about the type of cancer and cancer treatments, as well as personal information, such as name, date of birth, sex, provincial health care number, and postal code. The ACR will contact all potential participants on behalf of the research team with a study information letter with the research team’s contact information. Potential participants will include patients diagnosed with any type of cancer, who completed their treatments at least 2 weeks prior and who reside outside the Calgary metropolitan region. The geographical criteria were chosen to access cancer survivors in urban and rural areas of Alberta that are distant from the University Health Center, as most mindfulness-based programs and studies have been mainly accessible for residents of the Calgary metropolitan region due to its proximity to the University Health Center. The ACR’s method of contact ensures patient privacy and provides patients the choice to participate in the study. Interested participants will then contact the research team, and then are further screened for inclusion criteria.

### Sample Size

In previous studies with face-to-face group MBCR, we have observed medium effects for symptoms of stress (measured by the Calgary Symptoms of Stress Inventory [C-SOSI]) as the primary outcome and expect similar effects in this study. Also, recent meta-analyses of MBIs and psychosocial stress outcomes have demonstrated a similar medium standardized effect size of Cohen *d*=0.5 for stress [34]; also, most MBI studies collect their primary outcome at 3 months postbaseline as most mindfulness interventions span 6 to 10 weeks [7,34]. For this study, we therefore used a medium effect size (*f*=0.25) to conduct an a priori sample size calculation using the software G*Power 3.1.9.3 developed and maintained by researchers at Heinrich Heine University Düsseldorf [35]. The other parameters for estimating the sample size included, a standard type-1 error rate, α=.05, and a low risk of type-2 error, power (1-β)=.95, to detect interaction effects between time points (baseline and 3 months postbaseline) and group (intervention and control) using a repeated measures analysis of variance with correlation among repeated measures assumed to be 0.5 and nonsphericity correction ε=1. The total required sample size reported by G*Power was 54 participants to detect such an interaction effect.

In addition, based on our previous experience with online and in-person MBI trials and previous app-based studies [10,18,20,34,36], we assumed approximately 20% attrition and 10% to 15% probability of missing data. Therefore, we oversampled for this study accordingly. Hence, the final total sample size we aim to recruit in this study is N=74, with n=37 in the immediate intervention arm and n=37 in the waitlist control arm. Please refer to Figure 2 for the study flowchart that describes estimated sample size calculations at each assessment.

### Randomization

Participants will be randomized by the study statistician, by generating participant ID numbers and group allocations for the entire study in advance using a random number generator program in SPSS. Block lists of randomized participant IDs will then be uploaded to the Research Electronic Data Capture (REDCap) randomization module, which will allow the study staff to provide immediate group allocation to participants after completion of consent procedures. Only the study statistician will develop the group assignments, which are locked by REDCap after upload, to prevent selection bias. Randomization will occur after the baseline assessment, and those in the immediate *Am* MBCS intervention group will get a text message and email containing the link to download the *Am Mindfulness* app, from the Android or Apple app store. The waitlist control group will be informed that they will need to wait for the intervention and will be contacted when they can download the *Am* *Mindfulness* app and start the intervention.

### Informed Consent Procedures

Informed consent will be obtained electronically through the secure, web-based app designed to support data capture for research studies, REDCap, which is supported by the technology team at the University of Calgary, Canada, where this research is being conducted. REDCap’s web-based app uses secure two-factor web authentication, data logging, and secure sockets layer encryption that ensures the security and confidentiality of private information for obtaining informed consent [37]. An email with a survey link will be sent to the participant. After the participant clicks on the link for the study from their email client, the first page of the REDCap survey for this study will open in a new window or tab and will contain the details of the informed consent form. Participants will check the box “Yes” that asks them whether they completely understood the terms of their voluntary participation in the study.

Participants will then actively provide electronic consent to the study by clicking on the “Agree” button, which will be preceded by stating that, “Clicking on the ‘Agree’ button below indicates that (1) you have read the informed consent information, (2) you voluntarily agree to participate, and (3) you are at least 18 years of age.” Participants will also have the option to opt out of the study by clicking the “Disagree” button, which will be followed by the statement, “If you do not wish to participate in the research study, please decline participation by clicking on the ‘Disagree’ button.”

Only those participants that click on “Agree” will be able to proceed with completing the rest of the questionnaires. Participants will be able to download and save a PDF version of the consent form for their records. Participants will enter their name, email, and cellphone number after completing the form. After participants provide their online consent, they will be asked to complete the baseline measures online on REDCap. Subsequently, participants will be sent a study welcome email, which will contain orientation material and instructional pictures and videos about how to use the *Am* app, and instructions for the 4-week intervention. In addition, the study staff will also conduct a study orientation phone call to guide participants to using the app with ease.

### Intervention

#### The Am Mindfulness-Based Cancer Survivorship Journey

*Am Mindfulness* (“Am”; second generation of the app, *Wildflowers Mindfulness*) supports a personalized mindfulness practice through guided meditations, audio lectures and discourses, reminders, a timer to facilitate self-guided meditation, journaling features, and psychobiometric recordings and feedback. The *Am* app is currently available in four languages (English, Mandarin, Dutch, and German) and can be viewed and downloaded from the Android and Apple app stores with the platform-agnostic link [38]. Within *Am*, study participants will be instructed to access the *Am* MBCS journey. *Am* MBCS has a total of 27 steps that include, audio-recorded lectures, guided meditations such as body scans, and writing an events journal; see Figure 1 to screenshots of the *Am* MBCS journey contents. The curriculum is based on our previous experience with the evidence-based MBCR program and related meditations, which is described under 5 discrete units of learning in Table 2. The content of the audio instruction by LC and MS is similar to that delivered in the in-person classes on these units, with examples specific to situations and symptoms common for people living with cancer. Common topics such as coping with pain, insomnia and fear of cancer recurrence are included.

Participants will be encouraged to participate in the app-based activities for 20 to 30 min every day, with a minimum of 4 days in a week, over a period of 4 weeks. To promote engagement with the app-based program, user data will be tracked confidentially (see the section on *Feasibility, Acceptability, Adherence, and Contamination*) and users with low engagement will be sent text messages to promote app usage, eg, “Please remember to listen to your *Am* MBCS mindfulness recordings for today.” Text messages will be delivered using the secure communications platform, Twilio [39]. Also, the research team will monitor participant engagement metrics on a daily basis, and low or nonengaged users will receive a phone call with support for problem solving. In addition, *Am Mindfulness* also sends users regular push notifications, which appear as motivational messages on the user’s mobile screen that may also promote engagement.

Patients using the app can also access the meditations on *Am* through the app’s “Library” feature, which contains all the meditations on the app indexed by author and name. Although some meditations are perpetually free, users need to pay for the full selection of guided sessions and biofeedback technology; therefore, participants in this study will be provided with a 12-month paid subscription to the *Am* app.

**Table 2:** App-based mindfulness-based cancer survivorship curriculum.

Unit #	Topics or focus of module	Meditation	Exercise
1	What is mindfulness; why mindfulness for cancer?; belly breathing exercise; introduction to Body Scan with focus on cancer-related changes in the body.	Body Scan (short)	Positive events journal
2	Mindful attitudes (nonjudgment, acceptance, nonattachment) in the context of cancer.	Mindfulness of breath and mindful movement	Negative events journal
3	Stress response; biology of stress, stress and cancer; link between inner narrative and chronic stress; sleeping well exercise.	Mini breathing exercises, mindful movement, and walking meditation	Symptoms of stress checklist or mapping stress on the body
4	Stinkin’ Thinkin’; maladaptive stories we tell ourselves; common cognitive distortions with cancer-related examples; coping with thoughts and fears of cancer recurrence.	Open awareness	Thought log
5	Introduction to guided imagery; using imagery to cultivate loving kindness toward the suffering of self and others.	Mountain meditation and compassion meditation Body Scan (long)	Intention or plan moving forward

### Am’s App-Based Stress Measurement

**Figure 3:**
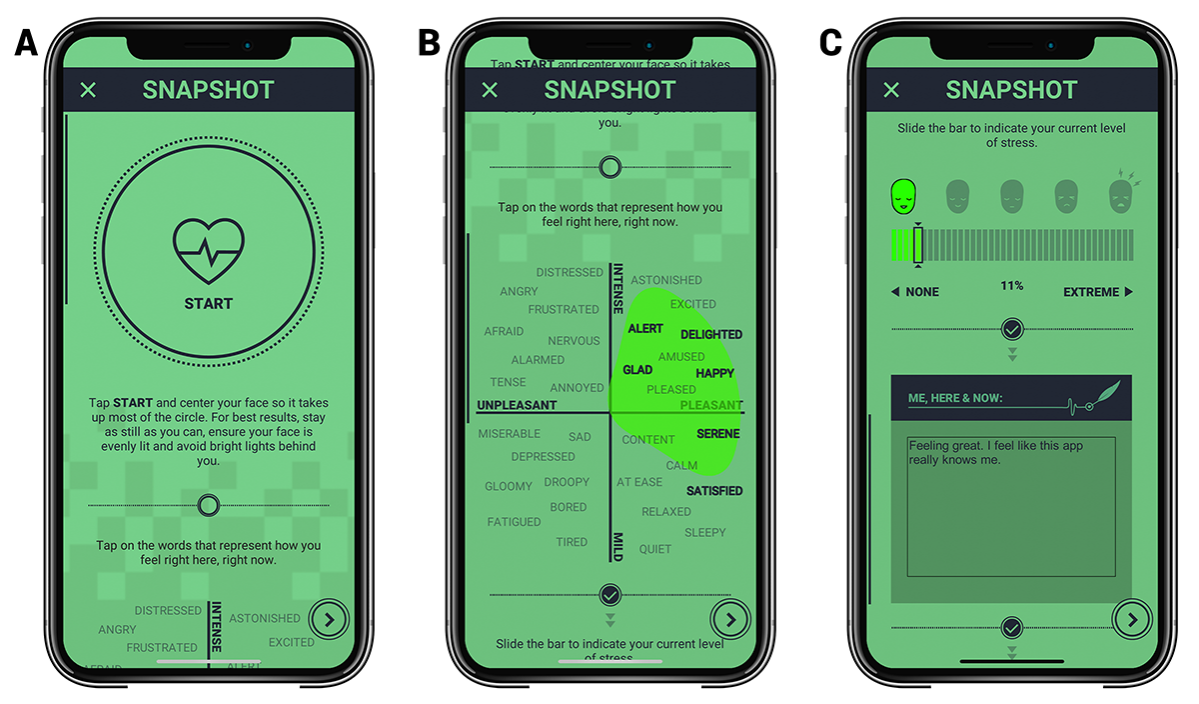
In-App Psychobiometric Assessments within *Am*. (A) “Selfie” video using photoplethysmography technology to quantify stress; (B) “Mood board” containing 32 emotion words and “stress slider” for stress self-assessment; and (C) “Journaling” feature to input experiences.

The *Am* app also has four innovative stress measurement features that serve as exploratory outcomes for this trial. First, cognitive stress is objectively quantified via a 30-second “selfie” video that uses an algorithm to extract heart rate and heart rate variability from the biosignals inherent to the human face using photoplethysmographic imaging principles [40]. The amount of cognitive stress is determined via deep neural networks trained on tens of thousands of video and stress pairings and has reported 86% accuracy for determining an individual’s stress as “very low,” “medium low,” “medium high,” and “very high” [41]. Second, emotional stress levels are obtained via a digital 4-quadrant emotion mapping board (mood board) that lists emotions such as “happy,” “sad,” and “tense” ranging on one axis from unpleasant to pleasant, and along another axis from mild to intense; see Figure 3 for screenshots of the in-app stress assessments. Each emotion listed within the mood board is associated with a score that is not disclosed to the user and used to calculate mood. Third, subjective stress is obtained via a slider ranging from “none” to “extreme.” Fourth, personal notes are input using an open field text box. The output of the mood board and stress slider provides data that have been benchmarked to standard psychological surveys [33].

The *Am* app uses secure web authentication, data logging, and encryption that ensures security and confidentiality of any personal identifiable information and in-app data*. Am* and its previous version *Wildflowers* have together received approximately 100,000 downloads and *Am* is rated 4.8 stars from 23 reviews in the Canadian Apple app store as of July 30, 2019.

### Procedures

#### Experimental Group

Participants will be provided a 12-month paid subscription to *Am*. The first month of this *Am* subscription is dedicated to the study intervention. Users will be encouraged to participate in the app-based activities for 20 to 30 min every day, with a minimum of 4 days in a week, after which they will get reminder notifications.

#### Waitlist Control Group

Participants will receive treatment as usual, followed by a delayed (waitlist) intervention of the same *Am* MBCS app-based intervention after the 3-month postbaseline assessment of the *Am* app intervention group (see Figure 2). Control group participants will get access to the *Am* app only after completing their 3-month waiting period.

### Trial Registration and Reporting

This trial has been registered at the ClinicalTrials.gov database of privately and publicly funded clinical studies [42]. The results will be reported as per the updated Consolidated Standards of Reporting Trials (CONSORT) eHealth checklist [43] version 1.6.1. and will follow guidelines and the flow diagram for reporting nonpharmacological treatments [44].

### Outcome Measures

The outcome measures employed in this study include a series of well-validated psychometric instruments for assessing a variety of psychosocial constructs. See Table 3 for a detailed description of outcomes. We will also use the standardized outcome measures available from the Patient-Reported Outcomes Measurement Information System (PROMIS), which is a set of person-centered measures that evaluate physical and psychosocial health in adults and children [45]. The advantage of using PROMIS measures is that they are psychometrically sound and have been created to be relevant across all conditions for the assessment of symptoms and functions [45]. Finally, we are also going to obtain and analyze the data from the *Am* app with regard to the user’s self-report, biometrics, and engagement. The entire battery of questionnaires will be completed securely online and requires approximately 30 to 45 min.

**Table 3:** Outcome measures.

Construct		Measure (abbreviation)	Description
**Screening measures**			
	Cognitive function	Brief Screen for Cognitive Impairment (BSCI) [46]	The BSCI consists of 3 items which are asked to the patient over the phone. The first item on the BSCI consists of a memory recall question, and the other 2 items ask about ability to carry out daily tasks without help. The scores obtained from the 3 items are then weighted and summed to arrive at the final BSCI score wherein >6 is significant impairment.
**Background measures**			
	Demographics and medical history	Age, sex, marital status, education, other medical conditions, and medications	Age, sex, marital status, education, other medical conditions, and medications. All these constructs will be assessed using standardized self-report items.
**Primary outcome**			
	Symptoms of stress	Calgary Symptoms of Stress Inventory (C-SOSI) [47]	The C-SOSI is a 56-item scale, derived from exploratory factor analysis on the 95-item Symptom of Stress Inventory (SOSI) collected from cancer patients who attended our MBCS program. A 5-point scale (“never” to “very frequently”) is used to rate the frequency of stress-related symptoms in the past week. There is a total score and 8 subscales (depression, anger, muscle tension, cardiopulmonary arousal, sympathetic arousal, neurological or GI, cognitive disorganization, and upper respiratory symptoms), all of which have high internal consistency (0.80 to 0.95), and the total score has good convergent and divergent validity with other well-validated measures.
**Secondary outcomes**			
	Fear of cancer recurrence	Fear of Cancer Recurrence Inventory (FCRI) [48]	FCRI contains 42 items, evaluating 7 components associated with the fear of cancer recurrence: triggers, severity, psychological distress and functioning impairments, insight scale, reassurance, and coping strategies. Each item is measure one a Likert scale ranging from 0 (not at all or never) to 4 (a great deal or all the time). Total score can be obtained from each subscale and a total FCRI score can be obtained by adding the total scores of all subscales, higher scores indicate higher levels of fear of cancer recurrence.
	Mindfulness	Mindfulness Attention Awareness Scale (MAAS) [49]	MAAS is a 15-item scale, designed to assess characteristics associated with mindfulness, such as open or receptive awareness of and attention to what is taking place in the present. Participants use a scale from 1 to 6 (almost always to almost never), to indicate how frequently or infrequently they have each experience. Higher scores reflect higher levels of dispositional mindfulness. A thorough validation process has demonstrated the reliability and validity of the MAAS with high internal consistency, α=.86.
	Rumination	Rumination-Reflection Questionnaire (RRQ) [50]	The RRQ is a 24-item, 5-point Likert Scale. The rumination subscale of the RRQ assesses recurrent, primarily past-oriented thinking about the self, which is prompted by threats, losses, or injustices to the self. The scale correlates with mindfulness in expected directions and has demonstrated high internal consistency of α=.92.
	Experiential avoidance	Acceptance and Action Questionnaire (AAQ) [51]	The AAQ was developed to measure experiential avoidance, the tendency to negatively evaluate internal experiences. (eg, emotions and body sensations), unwillingness to be in contact with such experiences, and the need to control or alter them or the contexts that engender them [51]. The psychometric properties of versions of the AAQ have been well established in clinical (eg, anxiety disorder) and nonclinical samples. The 16-item AAQ that will be used in this study produces a single factor, with acceptable internal consistency, α=.77.
	Anxiety	Patient-Reported Outcomes Measurement Information System (PROMIS)-Cancer Bank v 1.0–Anxiety [52]	PROMIS-Anxiety questionnaire assesses the anxiety domains of self-reported fear (fearfulness, panic), anxious misery (worry, dread), hyperarousal (tension, nervousness, restlessness), and somatic symptoms related to arousal (racing heart, dizziness). All PROMIS-Cancer instruments were developed for use with any cancer patient. The PROMIS-Cancer Anxiety item bank contains a total of 22 items, 20 of which are also in the PROMIS-Anxiety item bank, so it can be correlated with studies of other clinical populations. The PROMIS-Cancer Anxiety item bank will be delivered to patients in this study. The PROMIS-Cancer Anxiety has demonstrated high internal consistency (Cronbach α>.9).
	Depression	PROMIS-Cancer Bank v1.0–Depression [53]	PROMIS-Depression questionnaire for cancer patients assesses the domains of depression, which include self-reported negative mood (sadness, guilt), views of self (self-criticism, worthlessness), and social cognition (loneliness, interpersonal alienation), as well as decreased positive affect and engagement (loss of interest, meaning, and purpose). Somatic symptoms (changes in appetite, sleeping patterns) are not included. The PROMIS-Cancer Depression item bank contains a total of 30 items, 23 of which are also in the PROMIS-Depression item bank, so it can be correlated with studies of other clinical populations. The PROMIS-Cancer Depression item bank will be delivered to patients in this study. The PROMIS-Cancer Depression has demonstrated high internal consistency (Cronbach α>.9).
	Fatigue	PROMIS-Cancer Bank v1.0–Fatigue [54]	PROMIS-Cancer Fatigue measure assesses a range of self-reported symptoms from mild subjective feelings of tiredness to an overwhelming, debilitating, and sustained sense of exhaustion that likely decreases one’s ability to execute daily activities and function normally in family or social roles. Fatigue is divided into the experience of fatigue (frequency, duration, and intensity) and the impact of fatigue on physical, mental, and social activities. The PROMIS-Cancer Fatigue item bank contains a total of 54 items, all of which are also in the PROMIS-Fatigue item bank and will be delivered to patients in this study. The PROMIS-Ca Fatigue has demonstrated high internal consistency (Cronbach α>.9) in numerous studies within cancer and other clinical populations [54].
	Physical Function	PROMIS-Cancer Bank v1–Physical Function [55]	PROMIS-Physical Function instruments measure self-reported capability rather than actual performance of physical activities. This includes the physical functioning, mobility as well as instrumental activities of daily living, such as running errands. The PROMIS-Cancer Physical Function has items specific to cancer patients and survivors. The PROMIS-Cancer Physical Function item bank contains a total of 45 items, 33 of which are also in the PROMIS-Physical Function item bank [55], and will be delivered to patients in this study. The PROMIS-Cancer Physical Function has demonstrated high internal consistency (Cronbach α>.9).
	Return to work	Employment, hours of paid work, ability to work, and rate of return-to-work at 12-months	Self-reported work status will be assessed at each time point including (1) current working status (working full-time; part-time; retired; short- and long-term disability; unpaid homemaker); (2) weekly hours of paid work; and (3) job type using a well-established job classification system. If applicable, participants will be asked at follow-up on what date they returned to paid work.
**Exploratory measures**			
	User self-report	Mood, stress, and intent for mindfulness	Stress: Adjusting a dynamic slider between the minimum score “no stress” and the maximum score “max stress.” Mood Board: Participant can select between 1 and 24 “mood words” that indicate how they are feeling, eg, angry, happy, elated, and sad.
	User biometrics	Heart rate, respiratory rate, and relative blood oxygen saturation	Photoplethysmographic imaging, which is the measurement of volumetric change observed via the selfie camera of the smartphone, provides data that can be used to infer user biometrics, such as heart rate, respiratory rate, and relative blood oxygen saturation.

### Feasibility, Acceptability, Adherence, and Contamination

#### Feasibility of Intervention

The ACR provides the specific number of potential participants contacted for the study across the province. The number of participants who were invited through the registry, those who contact the team showing interest, as well those screened for eligibility, completion of intervention, and each assessment point will be tracked (see Figure 2). Intervention response rate and overall completion rate will be calculated from the aforementioned data points.

#### Acceptability, Adherence, and Engagement

The app usage of patients will be tracked through the engagement data from the app, which include session length, identity, type and frequency, points and badges earned, number of page and screen views, mindful activities in the app, total time spent on the mindfulness audio tracks, and number of daily visits to the app. *Am* also records which lessons and meditations participants accessed frequently. Participants with low engagement during the *Am* MBCS intervention period, defined as less than 4 times a week, will get reminder notifications through text message and phone calls with resources for problem solving. Acceptability and adherence to the intervention will be estimated from the engagement obtained from the app and completion rate of outcome assessments

#### Contamination

A standardized form assessing the use of a range of complementary therapies will be administered at each time point. We will also ask the waitlist control group if they used any other mindfulness or meditation apps during their 3-month waiting period.

### Objectives and Hypotheses

#### Objective 1: Primary Outcome

The first objective is to evaluate the efficacy of the *Am* app–based MBCS program to relieve symptoms of stress (primary outcome).

*Hypothesis 1:* Compared with controls, the *Am* app–based MBCS program participants will report significantly less symptoms of stress at 3 months postbaseline (primary outcome) assessment.

#### Objective 2: Secondary Outcomes

The second objective is to evaluate the efficacy of the *Am* app–based MBCS program to decrease the fear of cancer recurrence, anxiety, depression, and fatigue, and to improve overall physical functioning (secondary outcomes) at 3 months postbaseline assessment.

*Hypothesis 2:* Compared with controls, the *Am* app–based MBCS program participants will report significantly less fear of cancer recurrence, rumination, experiential avoidance, anxiety, depression, and fatigue, and increased mindfulness and overall physical functioning at 3 months postbaseline (secondary outcomes).

#### Objective 3: Exploratory Outcomes

This includes the exploratory aims as follows: (1) to explore correlations between the self-reported outcome data and the psychobiometric data collected by the *Am* app, (2) to determine changes over time between the *Am* app–based MBCS program and the waitlist control group.

*Hypothesis 3:* Self-reported data obtained from all participants will significantly correlate with the app-based self-reported stress data and biometric stress data.

*Hypothesis 4*: To determine the short-term, medium-term, and long-term effects of the *Am* app–based MBCS program with regard to the primary and secondary outcomes collected at all time points.

### Data Analysis

Participants will enter data from their home computers or smartphones using the secure REDCap data collection and management system (approved by the University of Calgary and Alberta Health Services). Data will then be transferred into SPSS and or SAS for analysis. Data analyses will utilize linear mixed models (LMM) and intent-to-treat (ITT) principles to assess several planned comparisons across the groups based on identified aims and hypotheses.

### Intent-to-Treat Analysis

Data related to recruitment, participation, and dropout rates will be reported according to the guidelines given by the CONSORT-eHealth statement [43]. All participants that entered our study will be included in our analyses and will be retained in the arm (treatment or control) to which they were originally randomly allocated. This study will employ an ITT analysis design, wherein participants who were nonadherent to the protocol will be included in statistical analyses, regardless of their alignment with the inclusion criteria, the treatment they received, and if they withdrew from the intervention protocol (attrition) completely or deviated from the protocol (nonadherence).

### Descriptive Statistics

Data will be cleaned, the descriptive statistics of the sample will be assessed, and all variables will be checked for normality of distribution. Descriptive statistics of our sample will be calculated to summarize demographic and disease-related characteristics and check for group differences between groups using Chi-squared and *t* tests. In case data are nonnormal, the Kenward-Roger correction for degrees of freedom will be applied to the LMM. Potential treatment moderators of age, sex, cancer type, cancer stage, and chemotherapy regimen will also be included as possible covariates. We will also conduct correlation and regression analyses to determine correlations between scores of the primary and secondary outcome measures, eg, correlation between C-SOSI and PROMIS-Cancer Anxiety scores, and between secondary outcome measures, eg, correlation between PROMIS-Cancer Fatigue scores and mood words selected by the participant in the *Am* app.

### Hypothesis Testing

#### Hypothesis 1

LMM is a suitable statistical method for this study because of the ability to perform sophisticated statistical imputation of data missing at random in a longitudinal study design. In addition, the LMM also includes mixed effect methods with a random intercept model, which can account for the variances between participants and within participants. Therefore, we plan to use the LMM analyses for testing hypotheses 1 and 2, wherein the LMM will estimate differences between the immediate group and waitlist control group by conducting a group × time interaction analysis with a significance level of α<.05. Each of the LMMs will include fixed effects for time (within-subjects factor) and group (between-subjects factor) and a random effect for the participant. Also, the restricted maximum likelihood estimate method in the LMM will be used to estimate the model parameters and standard errors with a compound symmetry covariance structure to account for the correlation between measurements. Data for testing hypothesis 1 will be C-SOSI total scores and subscale scores for 3 months postbaseline. Within- and between-group differences for the immediate and waitlist groups revealed by the LMMs will be reported with respective *P* values and the specific model effects, *F* (*df*).

#### Hypothesis 2

Similar to hypothesis 1, for hypothesis 2, we will use the same LMMs to test for within- and between-group differences for the secondary outcomes.

#### Hypothesis 3

For hypothesis 3, linear and curvilinear multiple regression models will be used, along with simple Pearson correlations to detect associations between the primary and secondary self-reported outcomes and the exploratory outcomes obtained from the app data.

#### Hypothesis 4

The short-term (2 weeks and 1 month postbaseline), medium-term (3 and 6 month postbaseline), and long-term (6 and 12 month postbaseline) longitudinal changes in the primary and secondary outcomes will be determined using the LMM quadratic model regressions. To account for the correlation between measurements, the restricted maximum likelihood estimate method in LMM will be used to estimate the model parameters and standard errors with a compound symmetry covariance structure. In addition, analyses with data nesting within participants will also be conducted that will control for the invariant part of each participant’s scores. The LMM regression weights (β) as well LMM regression coefficients will be reported along with a quadratic regression graph including all time points of data collection.

## Results

Recruitment commenced in January of 2019 and the target sample for enrollment was reached on May 2, 2019. Currently 83 patients have consented and enrolled in the study and are in various stages of their assessments and programs. Anticipated date for the completion of primary outcome data collection is August 1, 2019. Also, data collection for the entire trial is expected to be completed by May 2020.

## Discussion

### Limitations

Considering app-based mindfulness interventions in cancer care are still in the early stages of design and testing, this study has certain design- and intervention-related limitations. First, regarding study design–related limitations, this trial included survivors of all cancer types and stages, which results in high levels of variability of symptoms and cancer-related side effects, which may impact the internal validity of the trial and mask treatment-related effects because of the intervention. However, as the ultimate aim of this research is to reach all cancer survivors regardless of geography, the inclusion criteria were intentionally kept broad to mirror the real-world usage. Second, in terms of the intervention, we selected a 4-week duration for the app-based mindfulness program based on a similar app-based study of a previous version of *Am Mindfulness*, called *Wildflowers*, with anxious college students [33]. This 4-week length of the app-based program may not be long enough for cancer survivors, especially those who are completely new to mindfulness meditation and related stress management techniques. Indeed, in-person mindfulness programs for cancer survivors have been between 6 and 9 weeks [8,11,56]. Future research into dose-response efficacy of app-based mindfulness interventions in the cancer population is needed to provide an evidence-based duration for app-based mindfulness programs in cancer care.

### Conclusions

This study has the potential to provide a large-scale delivery tool for mind-body therapies to effectively reach cancer patients and survivors the world over. Cancer patients are often unable to successfully participate in face-to-face group programs for a variety of reasons. A smartphone app–based mindfulness program can overcome several difficulties faced by cancer survivors with participating in mindfulness interventions. Patients can participate from home in real time without the added burden of travel, parking, and walking to classes. If effective, this type of low-cost, mobile app–based intervention would be readily welcomed by patients and could easily be translated into clinical practice to reach a large number of patients and survivors, no matter where they reside, including those in remote locations.

## Abbreviations

ACR: Alberta Cancer Registry
CONSORT: Consolidated Standards of Reporting Trials
C-SOSI: Calgary Symptoms of Stress Inventory
ITT: intent to treat
LMM: linear mixed model
MBCR: Mindfulness-Based Cancer Recovery
MBCS: Mindfulness-Based Cancer Survivorship
MBI: mindfulness-based interventions
MI: Mobio Interactive Inc
PROMIS: Patient-Reported Outcome Information System
REDCap: Research Electronic Data Capture
